# Case Report: The importance of genetic counseling for families with hyperinsulinism

**DOI:** 10.3389/fped.2024.1520871

**Published:** 2025-01-17

**Authors:** Victoria R. Sanders, Katherine Lord, Winnie M. Sigal, Heather McKnight, N. Scott Adzick, Lisa J. States, Tricia Bhatti, Diva D. De Leon

**Affiliations:** ^1^ Division of Endocrinology, Children’s Hospital of Philadelphia, Philadelphia, PA, United States; ^2^ Congenital Hyperinsulinism Center, Children’s Hospital of Philadelphia, Philadelphia, PA, United States; ^3^ Department of Pediatrics, Perelman School of Medicine at the University of Pennsylvania, Philadelphia, PA, United States; ^4^ Department of Surgery, Children's Hospital of Philadelphia, Perelman School of Medicine at the University of Pennsylvania, Philadelphia, PA, United States; ^5^ Department of Radiology, Children's Hospital of Philadelphia, Perelman School of Medicine at the University of Pennsylvania, Philadelphia, PA, United States; ^6^ Department of Pathology and Laboratory Medicine, Children's Hospital of Philadelphia, Perelman School of Medicine at the University of Pennsylvania, Philadelphia, PA, United States

**Keywords:** hyperinsulinism, genetic counseling, carrier testing, *ABCC8*, recurrence risk

## Abstract

Congenital hyperinsulinism (HI) is the most common cause of persistent hypoglycemia in infancy. Genotype-phenotype correlations directly inform medical care for patients. Understanding the genetic etiology also allows accurate genetic counseling to be provided, illustrated by two families following a diagnosis of HI. A newborn had hypoglycemia at birth and was diagnosed with focal HI due to a paternally inherited recessive *ABCC8* variant. Years later the paternal half-sibling was diagnosed with HI. Testing revealed compound heterozygous *ABCC8* variants, consistent with diffuse disease. Following testing, the father's partner(s) should have been offered carrier testing. However, the parents were unaware that future children could be at increased risk of HI. The second family's son was diagnosed with HI in infancy and genetic testing identified a heterozygous recessive *ABCC8* variant. Parental testing revealed both parents carried this variant. Focal HI was subsequently confirmed. This family's 1/4 chance to have a child with diffuse HI was significantly higher than the 1/540 chance their child could have focal HI. Understanding the etiology of a patient's HI not only allows for appropriate medical management but has important reproductive implications for the family. Genetic counseling is an important component of the multidisciplinary care received by every family with HI.

## 1 Introduction

Congenital hyperinsulinism (HI) is the most common cause of persistent hypoglycemia in infancy, with an estimated incidence of around 1/28,000–1/50,000 live births (1–4). Over 30 genes have been identified to date as causes of HI, including genes for isolated HI as well as HI associated with multiple genetic syndromes (5). Even so, the genetic cause remains elusive in 21% of people with HI (6). The phenotype of HI is variable, regarding severity, triggers of hypoglycemia, and responsiveness to diazoxide, the only drug with regulatory approval for this indication. There are several histological subtypes of HI, including diffuse, focal and atypical pathology (7, 8). Diffuse HI, due to germline variants in associated genes, affects the entire pancreas and is characterized by β-cell nucleomegaly (9). Focal HI, which is diazoxide-unresponsive (6), results from somatic maternal loss of heterozygosity of chromosome 11p15 coupled with a paternal recessive variant in *ABCC8* (OMIM 600509) or *KCNJ11* (OMIM 600937) (10), causing an adenomatous hyperplastic lesion within the pancreas (9). Atypical histology, also referred to as Localized Islet Nuclear Enlargement (LINE) or mosaic HI, is associated with diazoxide-unresponsive HI due to mosaic variants in *ABCC8* and *GCK* (OMIM 138079) with histology similar to diffuse HI but restricted to only part of the pancreas (8). Management differs between the different subtypes of HI (11) (Table 1).

**Table 1:** K_ATP_ channel molecular pathways in hyperinsulinism.

K_ATP_ channel variants^a^	Diazoxide responsiveness	Mode of inheritance	Histology
Recessive	Unresponsive	Paternally inherited recessive variant with somatic loss of heterozygosity for the maternal chromosome 11p region → paternal isodisomy	Focal disease
		Biallelic recessive variants	Diffuse disease
Dominant	Unresponsive	Autosomal dominant variants	Diffuse disease
		Somatic autosomal dominant variants	LINE histology
	Responsive	Autosomal dominant variants	Diffuse disease

Adapted from (12).

^a^ K_ATP_ channel genes are *ABCC8* and *KCNJ11*.

Results of genetic testing can directly inform medical care for patients due to the strong genotype-phenotype correlations for several of the most common causes of HI (6, 11). For example, a paternally inherited *ABCC8* or *KCNJ11* recessive variant has a 94% positive predictive value for the presence of a focal pancreatic lesion in someone with diazoxide unresponsive HI (6). This result therefore indicates the need for imaging with 18-F-L 3,4-dihydroxyphenylalanine positron emission tomography (18 F-DOPA PET) scan to confirm the presence and location of a lesion, which can then be surgically excised, resulting in a cure when the lesion is completely removed (13, 14). In addition to the implications for management, understanding the genetic etiology also allows accurate genetic counseling to be provided to the family. Depending on the genetic etiology, HI could be due to autosomal recessive, autosomal dominant or rarely X-linked variants (6, 15), in addition to chromosome and imprinting defects (as observed in patients with Turner syndrome or Beckwith Wiedemann syndrome, respectively) (16, 17). While some forms of HI are inherited from affected or unaffected/carrier parents, other genetic alterations occur *de novo* in the child (6). Because of the complex nature of the potential genetic etiology of a given patient's HI, genetic counseling is an important component of the multidisciplinary care these patients and families require. We present two families affected by HI to illustrate the importance of providing comprehensive genetic counseling to families following a diagnosis of HI.

## 2 Case report

**Figure 1:**
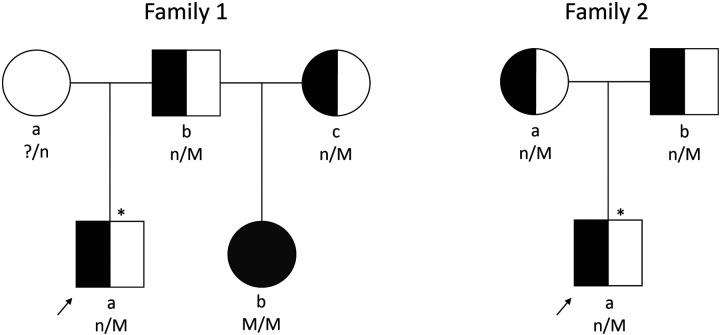
Pedigrees of two families described. Arrows indicate probands. Squares depict males and circles depict females. Filled symbols indicate diffuse HI. Half-shaded symbols indicate carrier of HI; empty symbol indicates unaffected with HI; * indicates focal HI; n/M, mutation carrier; n/n, mutation negative; ?/n, carrier status unknown; M/M, two mutations.

The first family (Figure 1) presented at an outside hospital with a newborn male who had persistent hypoglycemia at birth and was ultimately diagnosed with HI. He was initially treated with a glucose infusion rate (GIR) up to 30 mg/kg/min, as well as diazoxide, hydrocortisone and octreotide. Patient was transferred to our Hyperinsulinism Center, and a focal lesion was identified on 18 F-DOPA PET scan. The focal pancreatic lesion was surgically removed following two pancreatectomies (10% pancreatectomy in total). A fasting test proved resolution of the HI prior to discharge. Subsequent genetic testing revealed a paternally inherited recessive *ABCC8* variant (c.4480C>T, p.Arg1494Trp, NM_001287174.1). Several years later the paternal half-sister of this patient was diagnosed with HI. She required a GIR of 15 mg/kg/min and was additionally treated with continuous feeds and intravenous glucagon. Genetic testing revealed compound heterozygous pathogenic/likely pathogenic *ABCC8* variants [c.4261C>T, p.Arg1421Cys, NM_001287174.1 (maternally inherited); c.4480C>T, p.Arg1494Trp, NM_001287174.1 [(paternally inherited)] and a third *ABCC8* variant [c.1063G>A, p.Ala355Thr, NM_001287174.1 (maternally inherited)] classified as likely benign. These results were consistent with diffuse disease. Parents were unaware of the maternal *ABCC8* variants prior to evaluation of their daughter. Patient was transferred to our Hyperinsulinism Center for further management and ultimately required a 98% pancreatectomy. Histology revealed scattered islet cell nucleomegaly throughout the pancreas consistent with diffuse disease. Genetic counseling was provided to the family.

The second family's (Figure 1) 5-month-old son presented to an outside hospital with seizure activity and a plasma glucose of 47 mg/dl (2.6 mmol/L). He was subsequently diagnosed with HI following results of critical laboratory studies and transferred to our Hyperinsulinism Center after failing a diazoxide trial. Genetic testing identified a single recessive *ABCC8* variant (c.4013G>A, p.Trp1338*, NM_000352.4). Parental testing revealed both parents carried this variant. 18-F-DOPA PET scan identified a possible exophytic focal lesion arising from the pancreatic tail which was subsequently removed (2% pancreatectomy). Histological exam of the resected lesion noted well-circumscribed proliferation of endocrine tissue with absent p57 immunohistochemical staining of endocrine cell nuclei within the lesion. A cure fast revealed resolving HI prior to discharge. Patient underwent another cure fast around 13 months of age which confirmed resolution of the HI. Genetic counseling was provided to patient's family.

## 3 Discussion

Genetic counseling is an important component of the care families with HI should receive. In the first family, the father was a known carrier of a recessive *ABCC8* variant following the diagnosis of his son with focal HI. Carrier testing for the father's partner(s) should have therefore been discussed (18). Following the daughter's diagnosis of diffuse congenital HI due to compound heterozygous recessive *ABCC8* variants, parents indicated they were unaware that future children might be at risk for HI after the first child's diagnosis of focal HI given the rare occurrence of focal HI. The carrier frequency for pathogenic *ABCC8* variants in people who are not of Ashkenazi Jewish background is relatively low [approximately 1/125–1/177, based on cited population frequency and *ABCC8* gene accounting for approximately 40%–45% of cases of HI (19)]. However, in this case if both partners are carriers, recurrence risk is dramatically altered. It increases from 1/540 for focal HI (20) to 1/4 for diffuse HI, of which this family was unaware.

The second family further illustrates why the mother's genotype should be considered when providing genetic counseling to families with focal HI. In this family, the 1/4 chance that the parents would have a child with diffuse HI was significantly higher than the likelihood they would have a child with focal HI (1/540). Despite the lower probability of focal HI for this couple who were unknowingly both carriers of a recessive *ABCC8* variant, their child presented with focal HI. In the absence of parental testing, the family may have assumed a low recurrence risk in future pregnancies as the family in the first case assumed. Indeed, this very scenario was reported by Valayannopoulos et al. (21) in a consanguineous family whose first child had focal HI and only the father had genetic testing for the *ABCC8* variant identified in the child. This family had a subsequent child with diffuse HI which was the result of a homozygous variant inherited from each parent. Our family as well as the family reported by Valayannopoulos et al. clearly demonstrate that the presence of focal HI in a child does not exclude the possibility that the child's mother is a carrier of a recessive variant in the same gene. We therefore recommend that mothers of children with focal hyperinsulinism have full gene analysis of the gene associated with focal HI in their child prior to the parents having additional children.

These cases underscore the importance of providing comprehensive genetic counseling to families of children with HI. While the patients referenced had focal or diffuse HI due to pathogenic *ABCC8* variants, congenital HI has multiple known monogenic causes as well as associations with multiple genetic syndromes. Understanding the etiology of a patient's HI not only allows for appropriate medical management of the patient (11) but has important reproductive implications for the parents and family. When this increased risk is not known, families may be surprised by having additional medically complex children (21); infants suffering from hypoglycemia may go undiagnosed for many days to months, leaving them vulnerable to the morbidities associated with chronic or severe hypoglycemia, including seizures, developmental delays and cognitive impairments (21–24), and there may be delays in diagnosis (25–27) or in referrals to specialists who care for and treat patients with HI (22). Couples that are aware of their increased risk of congenital HI may defer having additional children or choose from multiple reproductive options including preimplantation genetic testing with *in vitro* fertilization, prenatal testing via chorionic villi sampling or amniocentesis, use of a gamete donor, adoption, or prompt diagnosis with glucose monitoring and genetic testing at birth. Genetic counselors are uniquely equipped to consider the implications of a genetic diagnosis for the whole family and can assist families and the medical team with the different considerations described above, including carrier testing for the mothers of children with focal HI and discussion of available reproductive options (28). Genetic counseling is therefore an important component of the multidisciplinary care received by every patient and family with HI.

## Data Availability

The original contributions presented in the study are included in the article/Supplementary Material, further inquiries can be directed to the corresponding author.
